# Bacterial communities of the cotton aphid *Aphis gossypii* associated with *Bt* cotton in northern China

**DOI:** 10.1038/srep22958

**Published:** 2016-04-15

**Authors:** Yao Zhao, Shuai Zhang, Jun-Yu Luo, Chun-Yi Wang, Li-Min Lv, Jin-Jie Cui

**Affiliations:** 1 State Key Laboratory of Cotton Biology, Institute of Cotton Research of CAAS, Anyang 455000, China; 2 Hubei Insect Resources Utilization and Sustainable Pest Management Key Laboratory, Huazhong Agricultural University, Wuhan 430070, China

## Abstract

Aphids are infected with a wide variety of endosymbionts that can confer ecologically relevant traits. However, the bacterial communities of most aphid species are still poorly characterized. This study investigated the bacterial diversity of the cotton aphid *Aphis gossypii* associated with *Bt* cotton in northern China by targeting the V4 region of the 16S rDNA using the Illumina MiSeq platform. Our sequencing data revealed that bacterial communities of *A. gossypii* were generally dominated by the primary symbiont *Buchnera*, together with the facultative symbionts *Arsenophonus* and *Hamiltonella*. To our knowledge, this is the first report documenting the facultative symbiont *Hamiltonella* in *A. gossypii*. Moreover, the bacterial community structure was similar within aphids from the same province, but distinct among those from different provinces. The taxonomic diversity of the bacterial community is greater in Hebei Province compared with in samples from Henan and Shandong Provinces. The selection pressure exerted by the different geographical locations could explain the differences found among the various provinces. These findings broaden our understanding of the interactions among aphids, endosymbionts and their environments, and provide clues to develop potential biocontrol techniques against this cotton aphid.

Aphids engage in symbiotic associations with a diverse assemblage of heritable bacteria. Aphid-associated bacterial community can vary with the sex and genotype of the insect host, and with environmental conditions, including temperature and diet^1^,^2^,^3^,^4^. Bacteria that are restricted to specialized insect cells and obligately vertically transmitted are known as primary symbionts^5^. Almost all aphids require the primary symbiont, *Buchnera aphidicola*, which provides nutrients not obtained in sufficient quantities from plant phloem^4^,^6^. Many phloem-sap-feeding insects also contain one to several other bacteria, called secondary or facultative symbionts, which may be localized to the bacteriocytes, other insect cells or the body cavity, and are capable of both vertical and horizontal transmission^7^.

While some aphids carry only the obligate symbiont *B. aphidicola*, most pea aphids, *Acyrthosiphon pisum*, are additionally infected with one or more facultative symbionts^8^,^9^. These symbionts confer various phenotypes to *A. pisum*, including defence against parasitism, protection against fungal pathogens, tolerance to heat stress, plant usage and reproductive manipulation^10^,^11^,^12^,^13^,^14^. Documenting the presence of facultative symbionts and identifying their effects on hosts can have important implications for the management of pest species.

*A. pisum* has been used as a model insect to study several questions related to the diversity and interactions of symbiotic bacteria with aphids^4^, but little information is available on the bacteria associated with other aphid species. High-throughput DNA sequencing approaches provide a new way to characterize bacterial communities, and this approach permits the investigation of ecological questions that could not be addressed using traditional methods^15^,^16^.

The cotton aphid, *Aphis gossypii*, has a worldwide distribution and causes damage to numerous economically important crops^17^. However, the bacterial communities harboured by *A. gossypii* are still poorly characterised. In the present study, we used the Illumina MiSeq platform targeting the V4 region of the 16S rDNA to determine (1) whether the bacterial communities in *A. gossypii* associated with *Bt* cotton were dominated by the primary and facultative symbionts, and (2) whether the diversity of the bacterial communities differed among multiple field populations of *A. gossypii* in northern China.

## Results

### Sequencing data

The Illumina MiSeq sequencing of the 16S rRNA gene amplicons from the field samples of the cotton aphid *A. gossypii* yielded 11,241–59,694 raw reads per sample (Table 1). After quality filtering and the removal of chimeric sequences, 10,710–57,034 reads per sample remained. The reads for the aphid samples could be assigned to 1358 OTUs, at 97% sequence identity. The rarefaction curve for every sample tended to saturation (Fig. S1), and the value of Good’s coverage of sequencing data in all samples was above 97% at the 0.03 dissimilarity cut-off (Table 1). These results indicated that our sequencing results captured most of the bacterial diversity associated with *A. gossypii*.

### Bacterial diversity in *A. gossypii*

Bacterial communities in the aphid samples were dominated by the phylum *Proteobacteria*, with a relative abundance of 95.56% (average values across all samples) (Fig. S2). In addition, *Proteobacteria* had four sub-phyla that dominated the aphid-associated communities (average abundance values across all samples): *Gammaproteobacteria* (90.47%), *Alphaproteobacteria* (2.57%), *Deltaproteobacteria* (1.75%) and *Betaproteobacteria* (0.68%) (Fig. S3).

At the family level, *Enterobacteriaceae* was the most dominant, with a relative abundance above 75% (Fig. S4). The relative abundances at the genus level are presented in Table 2. All aphid samples bore the primary symbiont *Buchnera* and two facultative symbionts, *Arsenophonus* and *Hamiltonella*. *Buchnera* dominated the bacterial communities in the cotton aphid *A. gossypii*. The least relative abundance of *Buchnera* was from the sample Cz, at 71.55%, and the highest was from the sample Jn, at 95.21%. The genera *Arsenophonus* was well-represented with relative abundances of 0.31%–4.74%. The relative abundance of *Hamiltonella* in all of the samples was less than 1%, except sample Dz. Furthermore, only samples from Cz, Sq, Bz and Dz bore the facultative symbiont *Wolbachia*, with relative abundances of less than 0.2%. Other facultative symbionts, such as *Regiella*, *Rickettsia*, *Serratia* and *Spiroplasma*, were not found in any of the collected samples (Table 2).

### Comparisons of bacterial communities from different provinces

**Figure 1:**
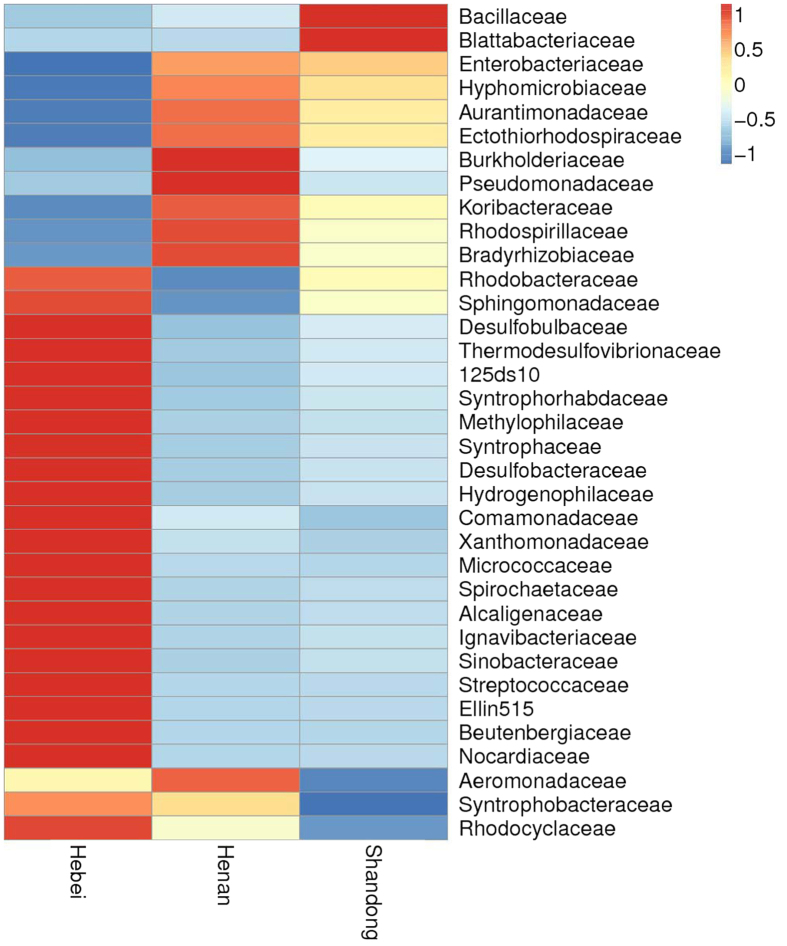
Heat maps showing the relative abundance and distribution of *A. gossypii* bacterial family in three provinces of northern China. The color code indicates relative abundance, ranging from blue (low abundance) to yellow to red (high abundance).

**Figure 2:**
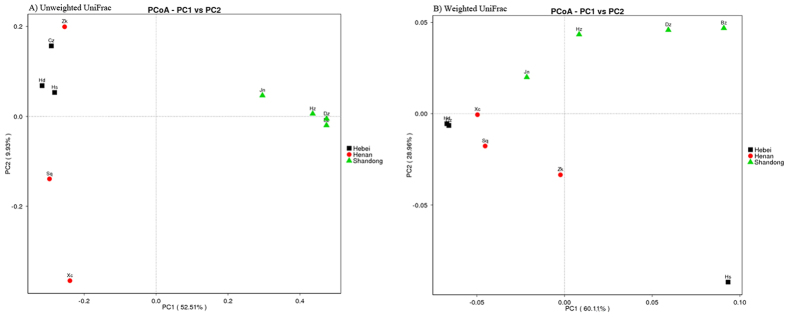
Comparison of bacterial community structures in *A. gossypii* samples in three provinces of northern China. Unweighted and weighted UniFrac metrics were used to determine pairwise distances between all samples.

The samples from Hebei Province were richer, having a higher number of operational taxonomic units (OTUs) than the samples from Henan and Shandong Provinces (Table 1). At the family level, the samples from Hebei generally also had more OTUs than those from Henan and Shandong, when the relative abundances of the top 35 OTUs were compared (Fig. 1). Additionally, the samples from Hebei had generally higher Ace and Chao1 richness estimates compared with the samples from Henan and Shandong (Table 1). Shannon and Simpson diversity indices also suggested that the taxonomic diversity of the bacterial community is higher in Hebei than in Henan and Shandong (Table 1). Principal coordinate analysis (PCoA) showed a distinct clustering among the individual samples, and the samples from same province tended to cluster together (Fig. 2).

## Discussion

Our sequencing data revealed that the bacterial communities in *A. gossypii* were generally dominated by the universally present primary symbiont *Buchnera*. Two facultative symbionts, *Arsenophonus* and *Hamiltonella*, were also found in all of the aphid samples. To our knowledge, this is the first report finding the facultative symbiont *Hamiltonella* in *A. gossypii*. Moreover, the bacterial community structure was similar within the same province, but distinct among different provinces (Fig. 2). Our results suggest that the bacterial diversity of *A. gossypii* is related to the geographical location.

To utilize phloem sap as their sole dietary component, most aphids are critically dependent on symbiosis with the bacteria *B. aphidicola*^6^. *Buchnera* was also the predominant genus found in the bacterial communities of our aphid samples. Previous studies have shown that facultative symbionts from five genera can infect *A. gossypii*: *Arsenophonus*, *Regiella*, *Rickettsia*, *Serratia* and *Wolbachia*^18^,^19^,^20^,^21^,^22^,^23^,^24^. Among these, *Arsenophonus* was found in all of our samples, *Wolbachia* was only detected in some of our samples, and they both had low relative abundances. The other three symbionts were not detected in our sequencing data. Brady *et al.* summarized the infection rates of *Regiella*, *Rickettsia* and *Serratia* in *A. gossypii*, and each of them was only 1%^25^. This may explain why these three facultative symbionts were not found in our results.

*Arsenophonus* is widespread in numerous insect species^26^. It can manipulate the reproduction of various parasitoid wasps by distorting the progeny sex ratio towards the production of females through male killing^26^,^27^. *Arsenophonus* may also behave as an obligate mutualist in hematophagous insects^28^, or as a facultative mutualist, protecting against parasitoid attacks in psyllids^29^. Jousselin *et al.* identified aphids as harbouring an important diversity of *Arsenophonus* strains, and the incidence was especially high in the *Aphis* genus^24^. Jousselin *et al.* also speculate that plant mediation and parasitism might be involved in the dispersal of *Arsenophonus*^24^. Moreover, *Arsenophonus* was reported to be involved in host plant specialization in the polyphagous aphid, *Aphis craccivora*^30^. In recent studies, *Arsenophonus* did not defend its aphid host *Aphis glycines* against major parasitoids and fungal natural enemies^31^, but provided a general benefit to *A. glycines*^32^.

*Wolbachia* is typically associated with manipulating the reproduction of several arthropod hosts, and it can rapidly reach a high frequency in host species as a consequence^14^,^33^. Additionally, *Wolbachia* has been implicated in providing a defence against viruses in other insects^34^, and it also can provide vitamin B to the host insect^35^. In our study, *Wolbachia* was only found in some of the aphid samples and their relative abundance was extremely low. Similarly, Liu *et al.* found a low titre of *Wolbachia* in *A. glycines*^36^. The detection of *Wolbachia* in aphids has some difficulties, which likely has resulted in it being under reported. One major difficulty is the current PCR protocols for the detection of *Wolbachia* were far from optimal^18^. In addition, the development of efficient *Wolbachia* detection was hindered by the presence of low titre infections and multiple infections^37^,^38^. *Wolbachia* density may be affected by co-infection with other *Wolbachia* strains or other vertically transmitted symbionts, as well as by host genotype^39^,^40^. Another difficulty in detection of *Wolbachia* is horizontal transfer of *Wolbachia* genes to host genomes^41^,^42^, which further complicates *Wolbachia* detection.

Surprisingly, *Hamiltonella*, which has not been reported in *A. gossypii*, was found in our data. *Hamiltonella* is known to protect aphids against parasitism. Oliver *et al.* found that *Hamiltonella defensa* reduced the rate of successful parasitism by *Aphidius ervi* by killing developing wasp larvae^43^. Multiple strains of *H. defensa* were examined in *A. pisum* and all of them conferred a partial protection against attack by *A. ervi*, indicating that *H. defensa* generally provides protection against this wasp^10^. In addition, parasitized *A. pisum* containing *H. defensa* produced significantly more offspring than parasitized uninfected aphids, indicating that *A. pisum* benefited from the *H. defensa* infection when under attack by parasitoids^44^. *H. defensa* being present in all of our samples may also suggest the importance of its role in protecting *A. gossypii* against parasitism in the field, and both vertical and horizontal transmission may act as drivers of *Hamiltonella* dispersal.

Many other bacterial taxa were also detected in some of the aphid samples, and their relative abundances were extremely low. For example, *Stenotrophomonas*, *Brevundimonas* and *Burkholderia*, which are commonly detected in environmental samples, were also found in our aphid samples^22^,^23^. *Burkholderia* is present in the environment, associated with insects and, in some instances, clearly acts as a mutualist^45^. Many of these bacteria could be contaminants. Additionally, our aphid samples from 10 different field locations could encounter different environmental factors. Although heritability cannot be ruled out, it is more likely that these bacteria engage in opportunistic associations with aphids (perhaps as gut associates or pathogens) or that they represent contaminants from soil, plants or human handling.

Geographical location and environmental factors may account for the different bacterial community structures found in aphids from different provinces. Natural populations of aphids may experience selection pressures from various management practices, natural enemies (pathogens, predators and parasitoids) and environmental conditions that could alter the composition, as well as the frequency, of associated bacteria^46^. In the PCoA, samples from the same province tended to cluster together (Fig. 2), suggesting that the samples might have experienced similar selection pressures.

In our results, the samples from Hebei Province had a higher number of OTUs, higher Ace and Chao1 richness estimates, and higher Shannon and Simpson diversity indices, compared with the samples from Henan and Shandong Provinces (Table 1). This may be because Hebei Province has a relatively higher latitude and may encounter different climactic variables, including temperature and precipitation. A geographical-based variation in infection frequencies has been reported for some facultative symbionts. In the pea aphid, *A. pisum*, symbiont prevalence was found to correlate with climactic variables^47^. Bacteria from the whitefly *Bemisia tabaci* differ in frequency based on the host plants and geographical locations^48^. Moreover, geographical variation in *Arsenophonus* symbiont prevalence was reported in the psyllid *Glycaspis brimblecombei*^29^. These studies suggest that environmental factors may act as important drivers of natural symbiont dynamics.

Here, we used the deep Illumina MiSeq sequencing of 16S rDNA genes, to analyse the bacterial communities of the cotton aphid *A. gossypii* associated with *Bt* cotton in northern China. Our sequencing data revealed that the bacterial communities of *A. gossypii* were dominated by the primary symbiont *Buchnera*, together with facultative symbionts that varied in incidence among the aphid samples. To our knowledge, this is the first report documenting the facultative symbiont *Hamiltonella* in *A. gossypii*. Selective pressures exerted by the geographical location could explain why the bacterial community structure was similar within the same province, but distinct among different provinces. These findings increase our understanding of the intricate symbiotic relationships between bacteria and *A. gossypii*. Further studies will be focused on identifying the functions of the representative bacterial species and determining whether these species could play important roles in the future as biocontrol agents.

## Methods

### Insect sampling and DNA extraction

**Figure 3:**
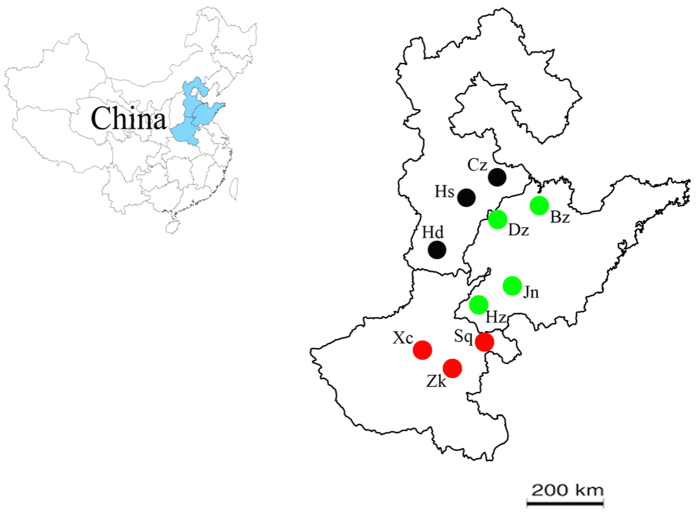
Sampling locations of *A. gossypii* in three provinces of northern China. Henan Province: Zhoukou(Zk), Xuchang(Xc), Shangqiu(Sq). Hebei Province: Cangzhou(Cz), Hengshui(Hs), Handan(Hd). Shandong Province: Jining(Jn), Bingzhou(Bz), Heze(Hz), Dezhou(Dz). The map is created with ArcGIS 10.2 (http://www.arcgis.com/features/).

Apterous adults of *Aphis gossypii* were collected from 10 field populations in northern China during August 2014 (Table 2 and Fig. 3). All of the sampling sites were planted with Cry1Ac cotton. The aphids were immediately immersed in 90% ethanol and frozen at −80 °C upon return to the laboratory.

Prior to DNA extractions, aphid samples, each comprising 20 adult aphids, were washed for 5 min in 70% ethanol and rinsed three times with sterile water to remove surface contaminants. Then, samples were hand-homogenized in extraction buffer (20 mM Tris-HCl pH 8.0, 2 mM sodium EDTA, 1.2% Triton^®^ X-100 containing 20 mg lysozyme ml^−1^). The homogenates were incubated at 37 °C for 40 min to achieve DNA extraction from both Gram-positive and Gram-negative bacteria. The DNA in the samples was then extracted using the TIANamp Genomic DNA Kit (TIANGEN Biotech (Beijing) LTD., China) following the manufacturer’s instructions. The quantity and quality of the DNA were measured with a NanoDrop 2000c spectrophotometer (Thermo Scientific, USA).

### PCR amplification, library preparation and sequencing

DNA was amplified using the 515f/806r primer set (515f: 5′-GTG CCA GCM GCC GCG GTA A-3′, 806r: 5′-XXX XXX GGA CTA CHV GGG TWT CTA AT-3′), which targets the V4 region of the bacterial 16S rDNA, with the reverse primer containing a 6-bp error-correcting barcode unique to each sample. PCR amplifications were carried out in a 30 μl mixture that included 15 μl of Phusion High-Fidelity PCR Master Mix (New England Biolabs, UK), 0.2 μM of forward and reverse primers, 10 ng of template DNA and nuclease-free water up to 30 μl. The PCR conditions were 98 °C for 1 min (1 cycle), 98 °C for 10 s, 50 °C for 30 s and 72 °C for 60 s (30 cycles), followed by 72 °C for 5 min. PCR products were mixed in equidensity ratios and mixture of PCR products was purified using the GeneJET Gel Extraction Kit (Thermo Scientific, USA).

Sequencing libraries were generated using a NEB Next Ultra DNA Library Prep Kit for Illumina (New England Biolabs, UK). The final quality and concentration of each library were checked using Agilent 2100 Bioanalyzer Instruments (Agilent Technologies, USA) and determined using KAPA Library Quantification Kits (Kapa Biosystems, USA). Sequencing was conducted on an Illumina MiSeq 2 × 250 platform at Novogene Bioinformatics Technology (Beijing, China) according to protocols described by Caporaso *et al.*^49^ and Kozich *et al.*^50^.

### Bioinformatics and statistical analysis

Paired-end reads were assigned to samples based on their unique barcodes and truncated by cutting off the barcode and primer sequence. Then, the paired-end reads were merged into single, longer sequences using FLASH (Version 1.2.7)^51^. Quality filtering on the raw tags was performed under specific filtering conditions to obtain high-quality clean tags^52^ according to the QIIME (Version 1.7.0)^15^ quality controlled process. Chimeric sequences were detected and removed using the UCHIME algorithm^53^.

Sequence analyses were performed using Uparse (Version 7.0.1001)^54^. Sequences with ≥97% similarity were assigned to the same OTU. Representative sequences from each OTU were screened for further annotation. For each representative sequence, the GreenGene Database^55^ was used with the RDP classifier (Version 2.2)^56^ to annotate taxonomic information. To study the phylogenetic relationships of different OTUs, and the differences in the dominant species of different samples, multiple sequence alignments were conducted using MUSCLE (Version 3.8.31)^57^.

To account for inequalities in sequence read depths among the samples, a randomly selected subset of 10,435 sequences per sample was chosen for a further bacterial community analysis. The microbial diversity was analysed using QIIME V1.7.0 and displayed with R software (Version 2.15.3)^15^. Alpha diversity analysis included observed species, Ace and Chao1 estimators, Simpson and Shannon diversity indices and Good’s estimate of coverage. A PCoA^16^ was performed to explore the differences in the bacterial community structures and was displayed with the WGCNA, stat and ggplot2 packages in the R software (Version 2.15.3). The sequencing data has been submitted to the NCBI database as a file under accession number SRP066541.

## Additional Information

**How to cite this article**: Zhao, Y. *et al.* Bacterial communities of the cotton aphid *Aphis gossypii* associated with *Bt* cotton in northern China. *Sci. Rep.* **6**, 22958; doi: 10.1038/srep22958 (2016).

## Supplementary Material

Supplementary Information

## Figures and Tables

**Table 1:** Sequencing analysis of 16S rRNA gene amplicons of *A. gossypii* with diversity indices.

Province	Sample	No.reads	No.OTU*	Ace	Chao1	Shannon	Simpson	Coverage
Henan	Zhoukou(Zk)	14,741	215	208.26	203.18	1.58	0.33	1.00
Henan	Xuchang(Xc)	58,353	223	243.41	195.81	1.13	0.26	1.00
Henan	Shangqiu(Sq)	11,241	107	124.70	119.33	0.88	0.19	1.00
Hebei	Cangzhou(Cz)	59,694	1034	1045.13	964.19	3.24	0.55	0.97
Hebei	Hengshui(Hs)	38,368	874	818.90	765.79	2.27	0.39	0.98
Hebei	Handan(Hd)	45,373	993	1093.36	975.18	3.02	0.49	0.97
Shandong	Jining(Jn)	44,998	248	240.37	211.67	0.68	0.13	0.99
Shandong	Bingzhou(Bz)	12,737	140	143.92	145.05	1.87	0.47	1.00
Shandong	Heze(Hz)	28,603	163	171.84	164.56	0.65	0.14	1.00
Shandong	Dezhou(Dz)	24,088	490	576.51	505.66	1.93	0.39	0.98

^*^Operational taxonomic units (OTUs) were defined with pairwise 97% sequence identity.

**Table 2:** Distribution and relative abundance of bacterial symbionts in *A. gossypii*.

Province	Site	Latitude/Longitude	Relative abundances (%)								
			*A*	*B*	*H*	*Re*	*Ri*	*Se*	*Sp*	*W*	Other
Henan	Zhoukou(Zk)	33°45′N, 114°27′E	2.70	83.06	0.14	0	0	0	0	0	14.10
Henan	Xuchang(Xc)	33°50′N, 114°14′E	4.74	87.93	0.11	0	0	0	0	0	7.22
Henan	Shangqiu(Sq)	34°31′N, 115°42′E	2.80	91.68	0.78	0	0	0	0	0.01	4.73
Hebei	Cangzhou(Cz)	37°58′N, 116°49′E	3.23	71.51	0.17	0	0	0	0	0.01	25.08
Hebei	Hengshui(Hs)	37°31′N, 115°39′E	0.31	84.73	0.20	0	0	0	0	0	14.76
Hebei	Handan(Hd)	36°47′N, 115°00′E	2.30	76.40	0.18	0	0	0	0	0	21.12
Shandong	Jining(Jn)	35°01′N, 116°18′E	1.40	95.18	0.10	0	0	0	0	0	3.32
Shandong	Bingzhou(Bz)	37°44′N, 117°39′E	2.24	73.57	0.68	0	0	0	0	0.01	23.50
Shandong	Heze(Hz)	34°47′N, 115°59′E	2.51	94.89	0.10	0	0	0	0	0	2.50
Shandong	Dezhou(Dz)	37°00′N, 116°00′E	0.99	84.09	7.07	0	0	0	0	0.19	7.66

^a^Facultative symbiont abbreviations: *A, Arsenophonus; B, Buchnera; H, Hamiltonella; Re, Regiella; Ri, Rickettsia; Se, Serratia; Sp, Spiroplasma; W, Wolbachia.*
