# Supplementary material for: Bacterial communities of the cotton aphid Aphis gossypii associated with Bt cotton in northern China
Source: Sci Rep. 2016 Apr 15;6:22958. doi: 10.1038/srep22958 (PMC4832190; doi:10.1038/srep22958)
Supplement: Supplementary Information [file srep22958-s1.pdf]

Supplementary Information

Bacterial communities of the cotton aphid *Aphis gossypii* associated with *Bt* cotton in northern China

Yao Zhao[1,2], Shuai Zhang[1], Jun-Yu Luo[1], Chun-Yi Wang[1], Li-Min Lv[1], Jin-Jie Cui[1]*

[1]State Key Laboratory of Cotton Biology, Institute of Cotton Research of CAAS, Anyang 455000, China, [2]Hubei Insect Resources Utilization and Sustainable Pest Management Key Laboratory, Huazhong Agricultural University, Wuhan 430070, China.

*Correspondence and requests for materials should be addressed to J.-J.C. (cuijinjie@126.com).

This Supplementary Information contains:

Supplementary Figure Legends

Supplementary Figures S1-S4

Supplementary Figure Legends

Figure S1. Rarefaction curve based on bacterial OTUs at a dissimilarity level of 3%. Henan Province: Zhoukou(Zk), Xuchang(Xc), Shangqiu(Sq). Hebei Province: Cangzhou(Cz), Hengshui(Hs), Handan(Hd). Shandong Province: Jining(Jn), Bingzhou(Bz), Heze(Hz), Dezhou(Dz).

Figure S2. Relative abundance of bacteria at phylum level in *A. gossypii* samples in three provinces of northern China. Henan Province: Zhoukou(Zk), Xuchang(Xc), Shangqiu(Sq). Hebei Province: Cangzhou(Cz), Hengshui(Hs), Handan(Hd). Shandong Province: Jining(Jn), Bingzhou(Bz), Heze(Hz), Dezhou(Dz).

Figure S3. Relative abundance of bacteria at class level in *A. gossypii* samples in three provinces of northern China. Henan Province: Zhoukou(Zk), Xuchang(Xc), Shangqiu(Sq). Hebei Province: Cangzhou(Cz), Hengshui(Hs), Handan(Hd). Shandong Province: Jining(Jn), Bingzhou(Bz), Heze(Hz), Dezhou(Dz).

Figure S4. Relative abundance of bacteria at family level in *A. gossypii* samples in three provinces of northern China. Henan Province: Zhoukou(Zk), Xuchang(Xc), Shangqiu(Sq). Hebei Province: Cangzhou(Cz), Hengshui(Hs), Handan(Hd). Shandong Province: Jining(Jn), Bingzhou(Bz), Heze(Hz), Dezhou(Dz).

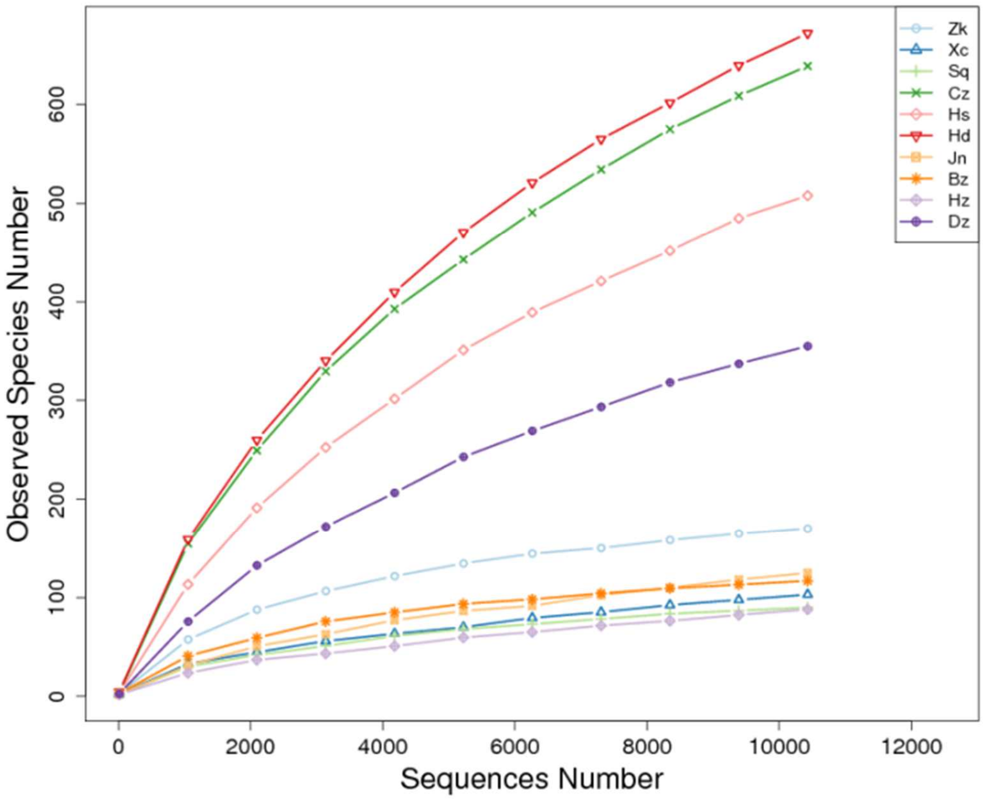
Observed Species Number
0
100
200
300
400
500
600
Sequences Number
0
2000
4000
6000
8000
10000
12000
Zk
Xc
Sq
Cz
Hs
Hd
Jn
Bz
Hz
Dz

Fig. S1

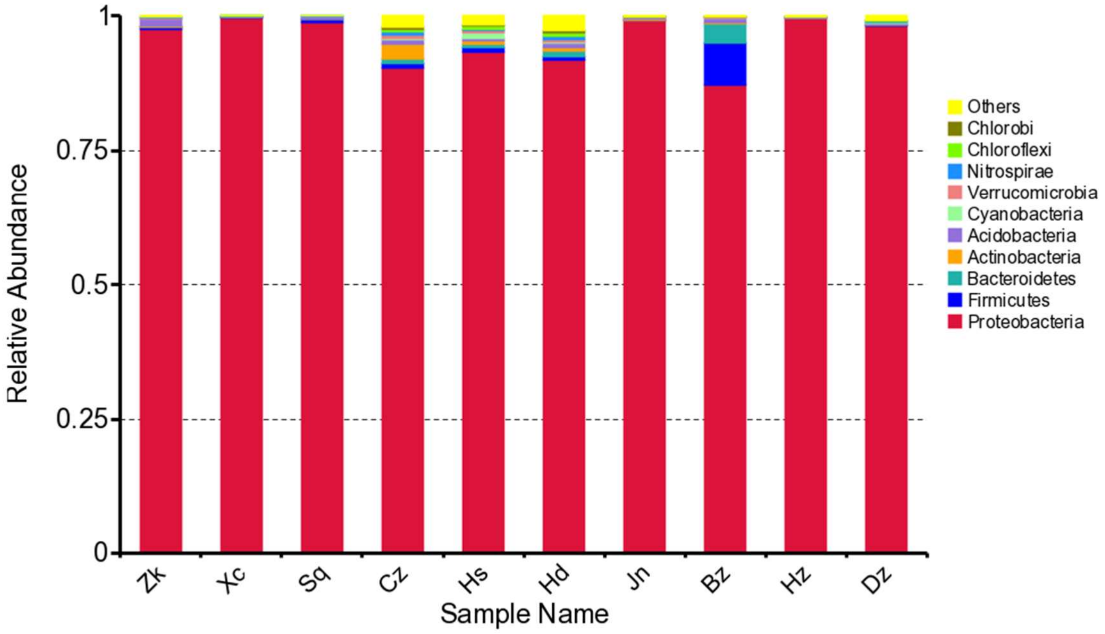
Relative Abundance
1
0.75
0.5
0.25
0
Zk
Xc
Sq
Cz
Hs
Hd
Jn
Bz
Hz
Dz
Sample Name
Others
Chlorobi
Chloroflexi
Nitrospirae
Verrucomicrobia
Cyanobacteria
Acidobacteria
Actinobacteria
Bacteroidetes
Firmicutes
Proteobacteria

Fig. S2

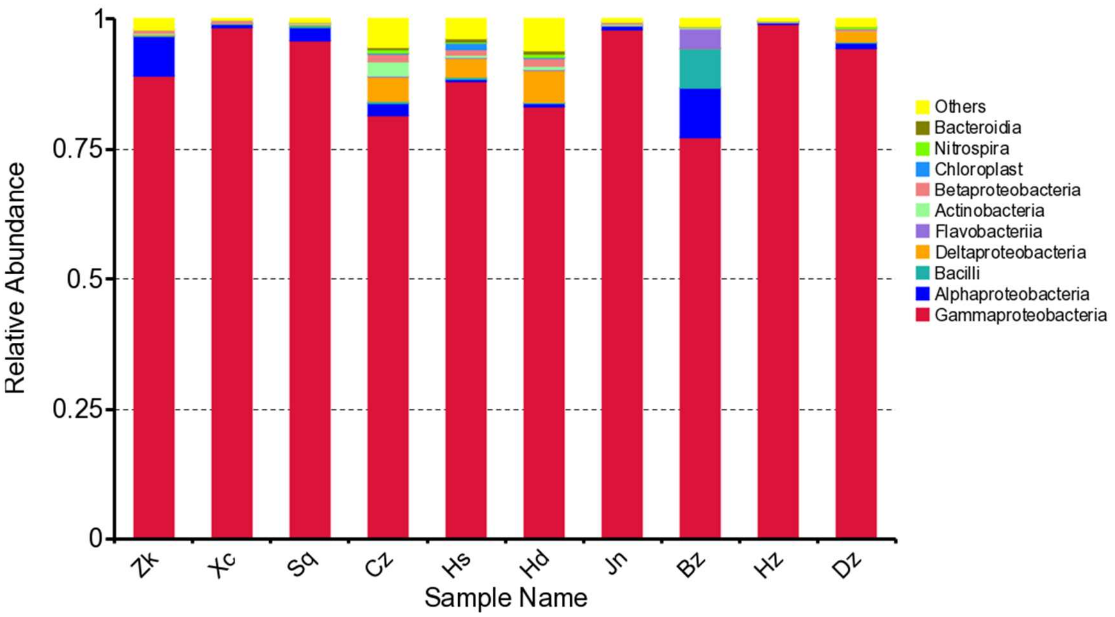
Relative Abundance
0
0.25
0.5
0.75
1
Zk
Xc
Sq
Cz
Hs
Hd
Jn
Bz
Hz
Dz
Sample Name
Others
Bacteroidia
Nitrospira
Chloroplast
Betaproteobacteria
Actinobacteria
Flavobacteriia
Deltaproteobacteria
Bacilli
Alphaproteobacteria
Gammaproteobacteria

Fig. S3

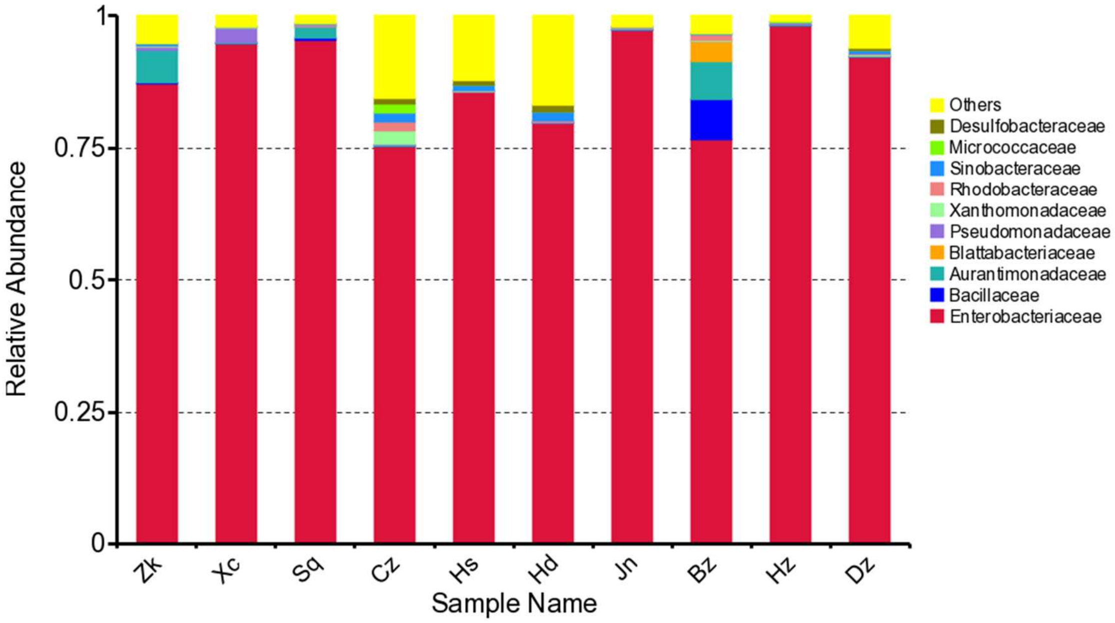
Relative Abundance
1
0.75
0.5
0.25
0
Zk
Xc
Sq
Cz
Hs
Hd
Jn
Bz
Hz
Dz
Sample Name
Others
Desulfobacteraceae
Micrococcaceae
Sinobacteraceae
Rhodobacteraceae
Xanthomonadaceae
Pseudomonadaceae
Blattabacteriaceae
Aurantimonadaceae
Bacillaceae
Enterobacteriaceae

Fig. S4
